# Extracellular vesicles derived from human ES-MSCs protect retinal ganglion cells and preserve retinal function in a rodent model of optic nerve injury

**DOI:** 10.1186/s13287-020-01702-x

**Published:** 2020-05-27

**Authors:** Seyedeh-Zahra Seyedrazizadeh, Sara Poosti, Abdoreza Nazari, Mehdi Alikhani, Faezeh Shekari, Farzad Pakdel, Koorosh Shahpasand, Leila Satarian, Hossein Baharvand

**Affiliations:** 1 Department of Brain and Cognitive Sciences, Cell Science Research Center, Royan Institute for Stem Cell Biology and Technology, ACECR, Tehran, Iran; 2 Department of Stem Cells and Developmental Biology, Cell Science Research Center, Royan Institute for Stem Cell Biology and Technology, ACECR, Tehran, Iran; 3 Ophthalmology Department, Eye Research Center, Tehran University of Medical Sciences, Tehran, Iran; 4 Department of Developmental Biology, University of Science and Culture, Tehran, Iran

**Keywords:** Human embryonic stem cells, Mesenchymal stem cells, Optic nerve crush, Extracellular vesicle, *Cis* P- tau

## Abstract

**Background:**

Retinal and/or optic nerve injury is one of the leading causes of blindness due to retinal ganglion cell (RGC) degeneration. There have been extensive efforts to suppress this neurodegeneration. Various somatic tissue-derived mesenchymal stem cells (MSCs) demonstrated significant neuroprotective and axogenic effects on RGCs. An alternative source of MSCs could be human embryonic stem cells (ES-MSCs), which proliferate faster, express lower levels of inflammatory cytokines, and are capable of immune modulation. It has been demonstrated that MSCs secrete factors or extracellular vesicles that may heal the injury. However, possible therapeutic effects and underlying mechanism of human ES-MSC extracellular vesicles (EVs) on optic nerve injury have not been assessed.

**Methods:**

EVs were isolated from human ES-MSCs. Then, ES-MSC EV was applied to an optic nerve crush (ONC) mouse model. Immunohistofluorescence, retro- and anterograde tracing of RGCs, Western blot, tauopathy in RGCs, and function assessments were performed during 2-month post-treatment to evaluate ONC improvement and underlying mechanism of human ES-MSC EV in in vivo.

**Results:**

We found that the ES-MSC EV significantly improved Brn3a+ RGCs survival and retro- and anterograde tracing of RGCs, while preventing retinal nerve fiber layer (RNFL) degenerative thinning compared to the vehicle group. The EVs also significantly promoted GAP43+ axon counts in the optic nerve and improved cognitive visual behavior. Furthermore, *cis* p-tau, a central mediator of neurodegeneration in the injured RGCs, is detectable after the ONC at the early stages demonstrated tauopathy in RGCs. Notably, after EV treatment *cis* p-tau was downregulated.

**Conclusions:**

Our findings propose that human ES-MSC EVs, as an off-the-shelf and cell-free product, may have profound clinical implications in treating injured RGCs and degenerative ocular disease. Moreover, the possible mechanisms of human ES-MSC EV are related to the rescue of tauopathy process of RGC degeneration.

## Introduction

Retinal ganglion cells (RGC) are one of the most important neural cells. Their axons make up the optic nerve and transfer visual signals to the brain. RGC degeneration due to direct physical trauma of the optic nerve (optic nerve crush; ONC), systemic inflammatory, or congenital or acquired diseases, such as glaucoma, can lead to blurred decrease of visual function and ultimately, blindness. Although various medical interventions that include neuroprotective medicines and surgeries have been widely employed to rescue neural cell damage, the outcome has not been promising [1].

Currently, mesenchymal stem cells (MSC) raise new hopes for treatment of retinal diseases and have been studied in many experimental models [2–4]. Notably, the therapeutic efficacy of MSC in models of ONC [5–9] and glaucoma [10–13] have been reported.

MSCs are frequently isolated from the bone marrow (BM), adipose and placental tissues, and umbilical cord blood (for review see [14]). These somatic tissue-derived MSCs have some drawbacks such as the need for a consistent source of cells and their low passage numbers. An alternative source of MSCs could be human pluripotent stem cells (PS-MSC) that include embryonic stem cells (ES-MSC) and induced pluripotent stem cells (iPS-MSC), with similar phenotypic and molecular characteristics that make them attractive candidates for regenerative cellular therapy (for review see [15]).

The therapeutic potentials of PS-MSCs in a variety of disease states have been demonstrated in many animal models [16–26]. Compared to somatic tissue-derived MSCs, PS-MSCs proliferate faster, express lower levels of inflammatory cytokines, and are capable of immune modulation [15, 24, 26, 27]. Interestingly, ES-MSCs were able to inhibit efficiently peripheral blood mononuclear cells (PBMCs), suggesting that ES-MSCs have a high immunomodulation activity [26]. Therefore PS-MSCs could be a promising cell source for regenerative medicine.

On the other hand, evidence strongly suggests the dominant mechanism of action of these cells is a paracrine-mediated effect with secreted factors. MSCs promote improvement of injured RGC through neuroprotective and neuritogenic cytokines and reduce inflammation with the help of anti-inflammatory and immunomodulatory properties (for review see [2, 28]). One effective paracrine-mediated mechanism could be through the secretion of bilayer membranous extracellular vesicles (EV), such as exosomes (40–100 nm in diameter) and microvesicles (0.1–1 mm in diameter) [29, 30] composed of proteins, growth factors, lipids, mRNAs, and miRNAs, which may possibly induce neural tissue regeneration through neuroprotective and neuritogenic effects [31]. The therapeutic efficacy of MSC-EVs has been demonstrated in many retinal disease models [32–40]. However, the long-term effect of PS-derived MSC-EV on RGC protection and function, as well as on p-tau abnormalities is unknown.

Tau is a phosphoprotein that is moderately phosphorylated under physiological conditions. Tau hyperphosphorylation results in its pathogenicity and neurodegeneration [41]. Accumulation of phosphorylated tau that has dissociated from microtubules may result in tau oligomers and tangles [42]. In particular, tau phosphorylation in the microtubule binding sites can affect microtubule dynamicity [43].

Various stress conditions can induce tau hyperphosphorylation, resulting in neurodegeneration [44]. Despite extensive considerations, the actual causative link between the physical damages and p-tau formation in RGCs has not been fully understood. In this regard, one major limiting drawback is the lack of observation of the early driver of tauopathy process upon the retinopathy. Recently, it has been demonstrated that phosphorylated tau at Thr-Pro motifs may result in two distinct *cis* and *trans* conformations. *Cis* to *trans* p-tau conversion is mediated by peptidyl-prolyl *cis*/*trans* isomerase 1 (Pin1) [45]. Pin1 suppression upon different stresses could result in *cis* p-tau accumulation [45, 46]. It has been claimed that *cis* p-tau conformers are almost pathogenic and prone to aggregation. Among several pathogenic species, we chose *cis* pT231-tau. We have reported that it is extremely neurotoxic and an early driver of tauopathy and the neurodegeneration process [47–49]. We have demonstrated that various stress conditions would reflect *cis* p-tau accumulation.

In the present study, we aimed to test the therapeutic potential of ES-MSC EV on an ONC mouse model. We sought to determine the effect of ES-MSC EV on optic nerve function and potential long-term neuroprotective effect by evaluating RGC survival, cognitive visual behavior, thickness of the retinal nerve fiber layer (RNFL), and *cis* p-tau accumulation.

## Materials and methods

### Mesenchymal stem cell (MSC) culture

Human ES-MSCs (passages 6 to 12) were provided from the Royan Stem Cell Bank. The provided MSCs had a spindle and homogenous morphology, confirmed expression of typical MSC markers, and were multipotent [26]. We have generated PS-MSC from different ESC and iPSC lines before, and they showed similar characteristics [24–26, 50]. Therefore, in here, we used one ESC line to generate MSC. The origin of human ES cell line was Royan H6 [51] (Royan Stem Cell Bank). The cells were cultured in Alpha-minimal essential medium (α-MEM, 11900073, Gibco) plus 10% fetal bovine serum (FBS, 10270, Gibco) and 1% l-glutamine (25030024, Gibco). We used two concentrations of FBS for different PS-MSCs, 15% [25] and 10% [24, 26], but we did not observe a significant difference in both concentrations. The culture medium was depleted from possible vesicles by ultra-centrifugation at 110,000*g* for 120 min. The medium was renewed every 3 days. The conditioned media were stored at − 70 °C until EV isolation.

### Extracellular vesicle (EV) extraction and quantification

To isolate EVs, ES-MSCs were cultured in T150 culture flasks up to 80% cell confluency (days 3–4). The EVs were extracted from the human ES-MSC culture medium using ultracentrifugation; all the centrifugation processes performed at 4 °C. First, the ES-MSC culture media were centrifuged at 3000*g* for 10 min; the resultant pellet was discarded. The supernatant was subjected to additional centrifugation at 20,000*g* for 30 min. The pellet was washed twice with phosphate-buffered solution without calcium and magnesium (PBS−, Gibco, 14190-136) and centrifuged again at 20,000*g* for 30 min. The supernatant was ultracentrifuged at 110,000*g* for 120 min, and the remaining pellet that contained the EVs was washed twice with PBS− and centrifuged at 110,000*g* for 120 min. The resultant vesicles were resolved in PBS and stored at − 70 °C. The EVs were thawed at 4 °C gradually for downstream tests. Then, the EVs were measured via dynamic light scattering (DLS, Zetasizer nano range), and the morphology was checked through scanning electron microscopy (SEM). Further, the enriched protein expressions (CD63, CD81, and TSG101) and a negative organelle marker (Calnexin) were checked by immunoblotting.

To reduce the batch effect of EVs, initially, we check out our process by a small batch. Then, we cultured cells in large-scale platform and EV isolation from pool of conditioned medium. Finally, the isolated EVs were characterized and aliquoted to be used in all experiments. By this approach, we used one EV batch which characterized for all experiments.

### Animals

C57BL/J6 male mice, approximately 8–10 weeks of age were kept on a 12-h day/night cycle. All procedures on the mice were in compliance with institutional guidelines and with the ARVO statement for the Use of Animals in Ophthalmic and Vision Research. The mice were anesthetized with a 1:4 mixture of xylazine/ketamine.

### The optic nerve crush (ONC) procedure

We used an operating microscope (Olympus, Tokyo, Japan) to generate a small incision in the conjunctiva beginning inferior to the left globe and around the eye temporally. With fine forceps (tweezers #5B forceps, World Precision Instruments), the exposed optic nerve was grasped approximately 1 mm from the globe for 5 s. During surgery, we applied a small amount of surgical lubricant to the eye to protect it from drying. At the end of the procedure, gentamycin (Daroupakhsh, Iran) and 1% tetracycline (Daroupakhsh, Iran) ointment were administered for postoperative infection control. The adequacy of the injury was histologically validated by Toluidine blue staining and by sacrificing additional animals 2 days after surgery [left (crushed) and right (intact) optic nerves].

### Experimental design

In addition to the age-matched intact group mice that did not receive any surgery, the model mice were randomly divided into three groups, 2 days after the surgery. The animals in the three groups received 200 μl infusions of either MSC (50,000), medium with no cells (vehicle), or EV (15 μg) [33, 34], via their tail veins. We evaluated different doses (250,000, 100,000, and 50,000 per injection) and found more survival rate after IV injection by 50,000 MSCs per injection (data not shown). The injections were performed every other day for three times per group for each animal. Therefore, totally, each animal received 45 μg EV or 150,000 MSCs. The systemic injections were repeated every other day for three times after the crush. EV concentrations were measured using the bicinchoninic acid assay according to the manufacturer’s instruction (Thermo Fisher Scientific).

### Cognitive visual behavior test: visual cliff

The visual cliff test was selected because the depth perception task depends on binocular vision and thus involves the primary visual cortex [52]. For the visual cliff test, we used a hand-made clear plastic box according to de Lima et al. [53]. In this box, the animal senses two shallow and deep sides because of the depth difference. The bottom of the box related to the deep area is suspended 70 cm above ground when the entire box is over a checkerboard. The animals were initially placed in the shallow area, and the time from first spotting until crossing the deep end (decision time) was recorded during a 2-min period. The videos from this cognitive visual behavior were evaluated by two investigators blinded to the experiment, *n* = 15 age-matched intact mice, *n* = 13 for vehicle group, *n* = 9 for MSC group, and *n* = 23 for EV group.

### Neural retina degeneration and immunostaining

We evaluated the neural retina survival rate as reported by Kurimoto et al. [54]. The eyes from the various mouse groups were enucleated on days 21 or 60 after the crush and placed overnight in 4% paraformaldehyde. Then, the front part of the eye sphere was cut and the retina was removed completely. After blocking the nonspecific background and to promote maximal immunoreactivity of monoclonal antibodies during immunoblotting with blocking solution, the whole retina was immunostained with anti-Brn3a, βIII tubulin (Tuj1), or *Cis* p-tau (gifted from Professor Lu), followed by the appropriate secondary antibodies (Supplementary Table 1). The presence of the MSCs in the retina was evaluated by human specific antibody for TRA-1-85. Images were captured using an IX71 fluorescent and confocal microscope (Zeiss LSM 800).

### Retrograde and anterograde tracing

In order to determine the amounts of intact and degenerated axons between the different groups, we examined retrograde and anterograde tracings. For the retrograde tracing, which is important for finding connections between the eyes and the brain, we injected 2 μl of 2% DiIC_18_ (3) (DiI; Molecular Probes, D282, UK) in each superior colliculus from the mice. The samples were harvested in 5–7 days. The harvested samples were fixed in 4% PFA for 1 h, mounted, and observed with a fluorescent microscope (IX71, Olympus, Japan). For anterograde evaluation, Chlorotoxin B (CTB; GenWay Biotech) was injected into the vitreous of the eyes. The mice were sacrificed 4–6 days after the injection. Longitudinal cryosections of optic nerves were created by Cryostat at 8 μm and treated by blocking solution for 1 h. The axons were immunostained against CTB or GAP43, and the images were captured with a fluorescent microscope.

### Western blot analysis

The extracted proteins from EVs were loaded on SDS-PAGE following by fixation and staining with Coomassie brilliant blue G-250 (Bio-Rad, Hercules, CA) and scanning by GS-800 densitometer (Bio-Rad).

Western blot was employed for expression analysis of *cis* p-tau, Tuj 1, and Pin 1 in the retinas along with optic nerves, as well as EV protein markers including three positive (CD63, CD81, and TSG101) and a negative organelle marker (Calnexin). Samples were deep frozen, and then the proteins were extracted by a Qproteome Mammalian Protein Prep Kit, according to the manufacturer’s instruction (Qiagen). The concentration of the proteins was determined via a Pierce BCA Protein Assay Kit, according to the manufacturer’s instruction (Thermo Fisher Scientific). For both tissue and EVs, 20 μg total protein from each replicate was separated on SDS-PAGE gels and transferred to PVDF membranes using a semi-dry electrotransfer system. The blots were then washed with tris-buffered saline that contained 0.1% Twin 20 for 15 min. Afterwards, blots were blocked with 2% skimmed milk for 1 h for tissue and by 5% BSA for EVs. Subsequently the blots incubated with primary antibodies (Supplementary Table 1, 1.5 h at RT for tissue, and overnight at 4 °C for EVs). After washing for three times, the blots were incubated with HRP-conjugated secondary antibodies, 1 h at RT for tissue, and 1 h at 4 °C for EVs. The protein bands were detected using ECL substrate (Thermo Scientific) and imaged by a chemiluminescence imaging system (Uvitec, Alliance Q9). The optical density (OD) of the bands from tissue blots was quantified by ImageJ software (https://imagej.nih.gov; National Institutes of Health, Bethesda, MD, USA), and ODs were normalized to β-actin for *Cis*-p tau, Tuj1, and Pin1.

### EV labeling and tracking

To trace EVs after intravenous injection in animals, they were labeled with a luminal fluorescent dye (Calcein-AM) as previously described [55]. Briefly, EVs were resuspended in 100 μL calcein AM solution and incubated at 37 °C for 20 min. Unincorporated Calcein AM was removed using exosome spin columns (MW 3000).

The presence of Calcein-EVs in the eye was visualized with UVI gel documentation (UVItec, Cambridge, UK) and analyzed with UVI photo version Q9 alliance software (UVItec, Cambridge, UK).

### Statistical analysis

Statistical differences were evaluated by ANOVA and the Tukey’s post hoc test or the unpaired *t* test. The data are presented as mean ± SD. *P* values < 0.05 were considered statistically significant.

## Results

### Characterization of extracellular vesicles (EVs)

**Fig. 1:**
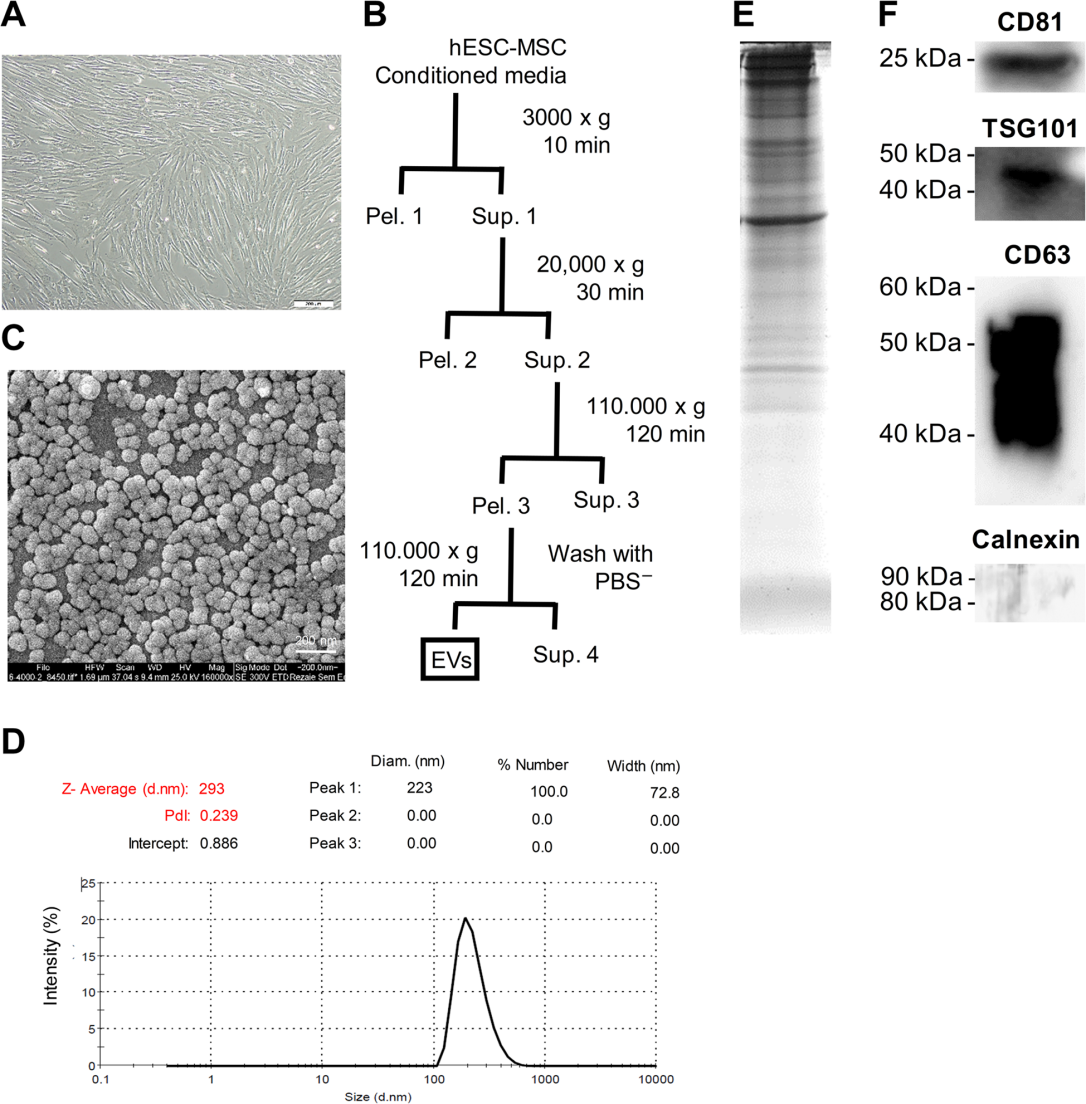
Isolation and characterization of embryonic stem cell-derived mesenchymal stem cells (ES-MSC) extracellular vesicles (EVs). **a** Phase contrast micrograph of passage-9 human embryonic stem cell-derived mesenchymal stem cells (ES-MSCs). **b** Extracellular vesicles (EVs) were isolated from conditioned medium of human ES-MSCs using differential ultracentrifugation. The supernatants (Sup.1–3) were submitted to further centrifugation and the pellets (Pel. 1–3) and Sup. 4 were discarded. **c** Scanning electron microscopy (SEM) of the EVs. **d** Size distribution of ES-MSC EVs was assessed using dynamic light scattering (DLS). Size quantification data demonstrated an average size of 130 nm for the EVs. **e** The Coomassie stained gel of loaded EVs. **f** Representative Western blot analysis for expression of enriched proteins in EVs (two EV membrane markers [CD63 and CD81], and one laminal marker [TSG101])

Human ES-MSCs were a homogenous population that had spindle-shaped morphology (Fig. 1a). To prepare EVs, the MSC conditioned medium was processed as shown in Fig. 1b. The final sediment that contained EV was resolved up to 100 μl of sterilized PBS and kept at − 70 °C. The spherical morphology of the EVs was demonstrated by SEM (Fig. 1c). The DLS assay verified that the EVs were approximately 220 nm in size (Fig. 1d). The protein pattern and concentration of isolated EVs has been confirmed by Coomassie staining of SDS-PAGE gel. Furthermore, the isolated EV were positive for the related markers CD81, TSG101, and CD63 as detected by Western blot analysis (Fig. 1f) and were negative for calnexin.

### Improvement of visual behavior after extracellular vesicle (EV) administration

**Fig. 2:**
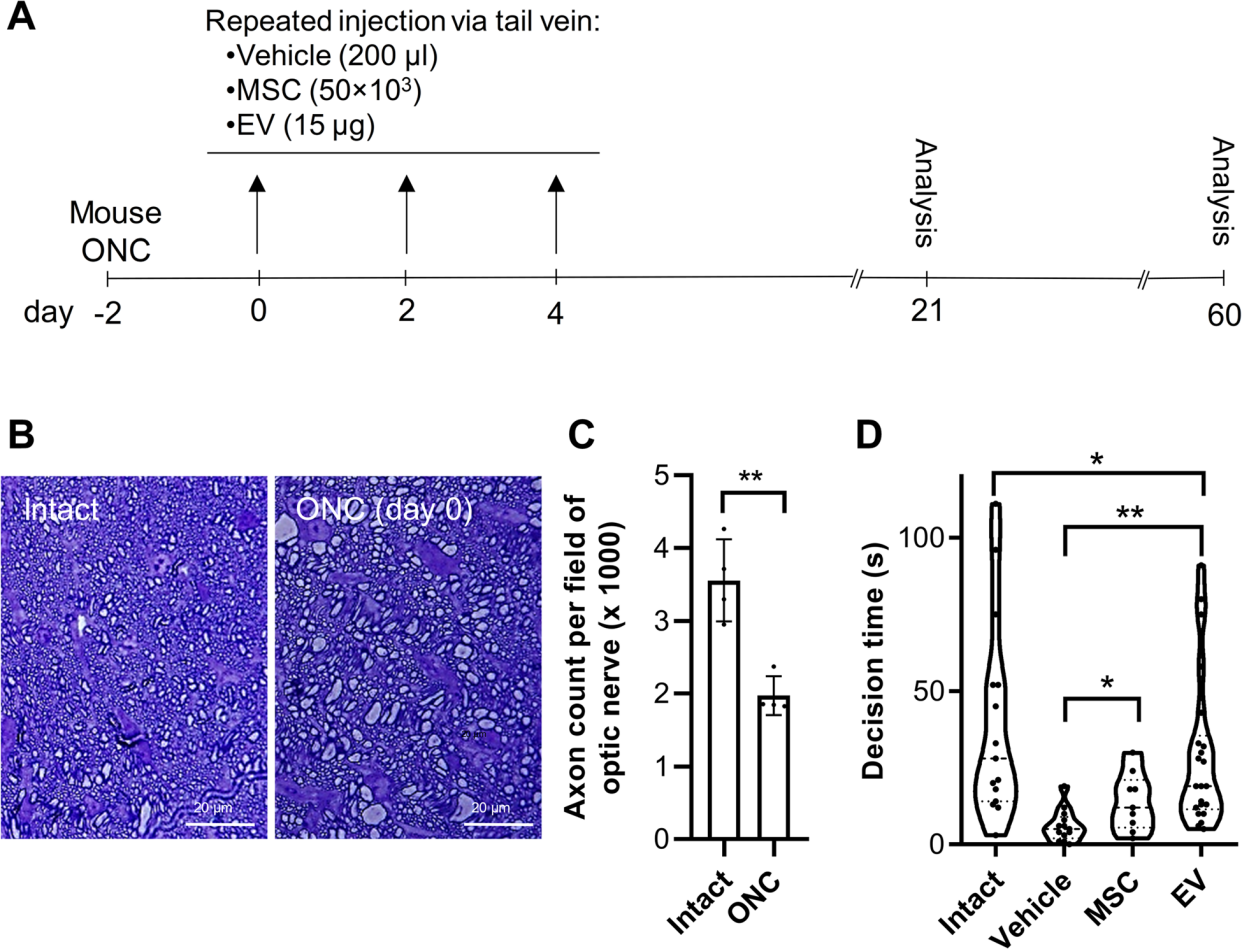
Animal groups and partial recovery of cognitive visually behavior. **a** Experimental design, time line, injection intervals, and analysis times. **b** Toluidine blue semi-thin optic nerve sections with optic nerve crush (ONC) and without crush (intact) which confirmed axonal loss during 2 days after the injury. **c** Quantification of toluidine blue semi-thin optic nerve sections in (**b**). The data demonstrated that more the 50% axons loosed 2 days after injury. Data presented as mean ± SD for four optic nerves per group. The data were analyzed by the un-paired *t*-test. **P < 0.01. **d** Decision time through visual behavioral test shows improvement of retinal function in the mesenchymal stem cell (MSC) and EV groups compared to the vehicle group 60 days post-treatment. Intact group animals are the same age as the healthy mice. Data are shown with a violin plot and analyzed by one-way ANOVA and post-hoc Tukey’s test. *P < 0.05, **P < 0.01

Since there are many reports of paracrine effects of MSCs on different injuries, we hypothesized that transplantation of the MSCs could improve the functional recovery in ONC of mice by administration of EVs. Two days after ONC, each mouse received either 50,000 MSCs, vehicle, or 15 μg EV every other day for three times (Fig. 2a). Toluidine blue staining of the semi-thin sections obtained from the optic nerve proximal to the crushed site after 48 h showed a distinct axon degeneration and reduced number of axons. We observed that more than 50% of the axons were lost and had myelin destruction (Fig. 2b and c). In order to evaluate functional consequences of regeneration and by taking advantage of the animals’ innate aversion to depth, we assessed depth perception using a visual cliff apparatus on day 60 post-injury. This test shows the connection between the optic nerve and the visual cortex. The mice first entered the shallow area, and we recorded their decision time, which was determined to be the time it took the mice to cross the deep border area after recognizing it. As shown in Fig. 2c, the average decision time was 39.53 ± 32.41 s for the intact, 6.07 ± 5.23 s for the vehicle, 13.88 ± 9.36 s for the MSC, and 29.27 ± 25.13 s for the EV groups. A significant improvement was demonstrated in MSC, and EV groups compared to the vehicle (at least *P* < 0.05). These results indicate that, under MSC or EV conditions, protecting the host RGCs leads to partial recovery (vision-driven behavior).

### Mesenchymal stem cells (MSCs) and extracellular vesicles (EV) protect retinal ganglion cells (RGCs)

**Fig. 3:**
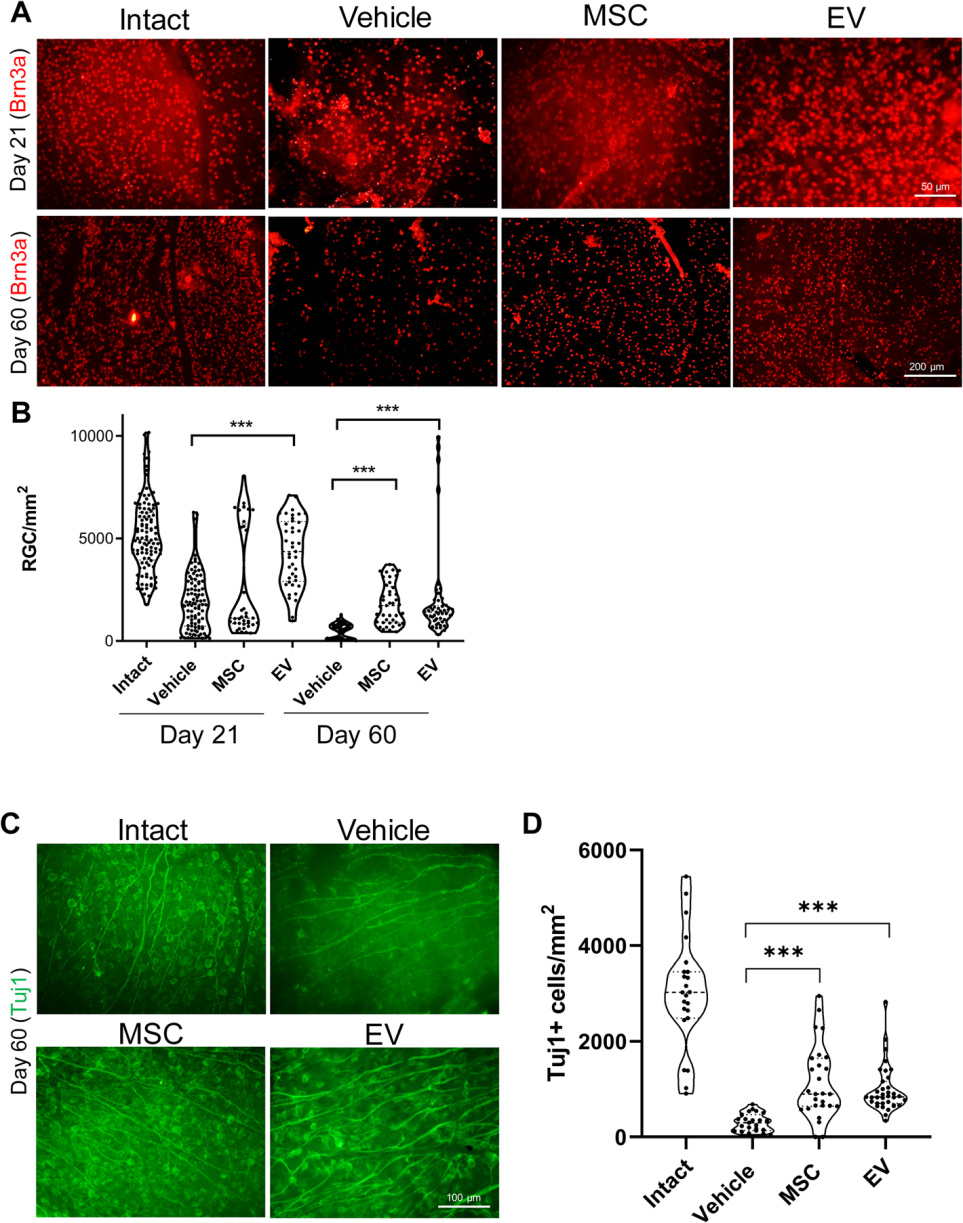
Retinal ganglion cell (RGC) survival at days 21 and 60 post-treatment. **a** Retinal ganglion cell (RGC) survival visualized by immunostaining for Brn3a. **b** Quantification of Brn3a+ cells by immunostaining in (**a**). Brn3+ cells were counted in 12 areas and averaged to estimate the RGC survival per mm^2^ in four to six optic nerves per group. Data are given as mean ± SD and analyzed by one-way ANOVA and post-hoc Tukey’s test. ***P < 0.001. **c** Immunostaining of retina for Tuj1 that shows the Brn3a expression trend

In an attempt to study the neurotrophic or neuroprotective effect of the treatments on ONC, we assessed the effects of MSC and EV injections on RGC survival. We examined the Brn3 marker to evaluate the effect of the treatments on RGCs degeneration in crushed mice retina at 21 and 60 days after the injury (Fig. 3a). The number of labeled RGCs within the retina was quantified and compared between groups (Fig. 3b). In animals that had intact visual system, the labeled cells had a density of 5128.00 ± 1783.89 RGCs/mm^2^. Three weeks after infusion of the vehicle, the number of labeled RGCs had reduced to 1979.78 ± 1434.05 RGCs/mm^2^ with an additional decrease after 60 days (487.19 ± 351.90 RGCs/mm^2^). In contrast, the MSC injections significantly protected the RGCs from neurodegeneration on day 21 (2708.21 ± 2598.60 RGCs/mm^2^) and day 60 (1809.25 ± 992.76 RGCs/mm^2^). The EV injections also had a significantly protective effect against neurodegeneration on day 21 (4321.49 ± 1653.20 RGCs/mm^2^) and day 60 (1996.28 ± 2215.81 RGCs/mm^2^). We examined Tuj1, another RGC-specific marker, on day 60 post-injury. We observed more Tuj1+ cells in the MSC- and EV-treated groups than the vehicle group (Fig. 3c).

These results demonstrated a significant improvement in decision time and RGC survival rate on day 60 post-injury in both MSC and EV groups in comparison with vehicle group. But there was not a significant difference between MSC and EV groups. Therefore, we continued our experiments with EV group.

**Fig. 4:**
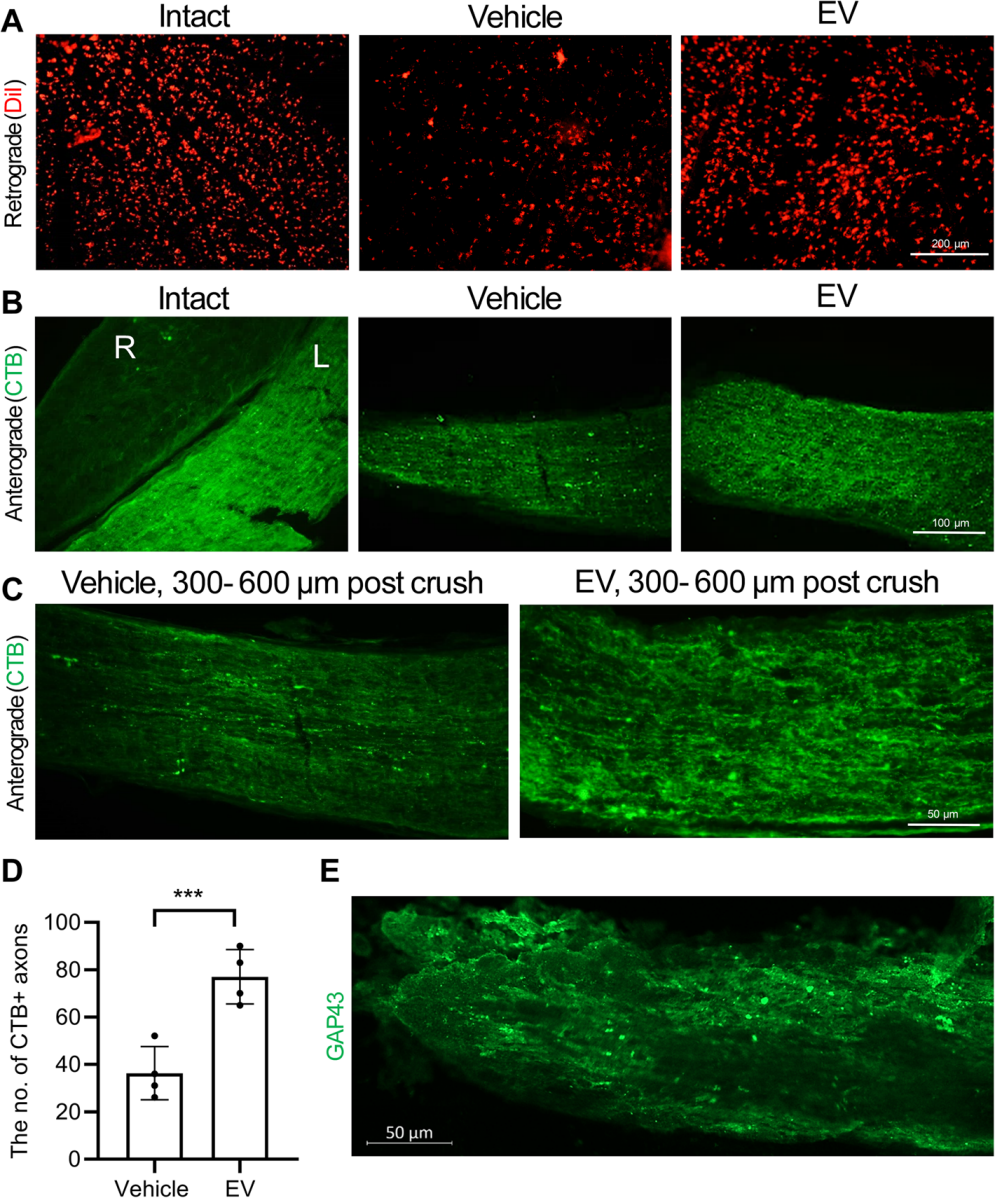
Retro- and anterograde axonal tracing 60 days post-treatment. **a** The intact retinal ganglion cells (RGCs) soma in the vehicle and extracellular vesicle (EV) groups that was retrogradely stained with DiIC18(3) (DiI; red). **b, c** Longitudinal cryosections and immunostaining against chlorotoxin B (CTB) in the intact group of the anterograde shows the left (L) and right (R) optic nerves. CTB was only injected in the left optic nerve. Data from the anterograde tracing shows that more CTB+ axons extended the length of the optic nerve in the EV group, whereas smaller numbers in the vehicle were seen, even at a distance of 300 to 600 μm in distal site of crush. **d** Quantification of CTB+ RGCs axons in the optic nerve in (**c**). Data are shown as mean ± SD for four optic nerves per group. The data were analyzed by the un-paired *t* test. ***P < 0.001. **e** A longitudinal section of the optic nerve with EV, which shows numerous axons with GAP43 expression

Next, we assessed the effects of the EV injections on RGC projections to the brain. A retrograde tracing approach that used microinjections of DiI into the superior colliculi and its detection within the RGCs layer of the retina was used. Our findings on the 60th day post-crush showed that the EV group had more regenerating axons than the vehicle group (Fig. 4a). In order to study the ganglion axonal integrity, we employed the anterograde tracing test using CTB, which passes through axonal terminals (Fig. 4b). The left eyes from the vehicle and EV groups received 3 μl of toxin in the vitreous while the right eyes were intact. The optic nerve was thoroughly harvested (without stretching). The left eyes that received CTB passed it along with their axons. The right eye from the intact group was used as the negative control. We also assessed 300 to 600 μm distal to crush area and found significant more CTB+ axons in this region in the EV group than the vehicle (Fig. 4c and d). More axons in the EV group stained positive for the growth-associated protein GAP43, as a marker of regenerated axons of RGCs (Fig. 4d).

**Fig. 5:**
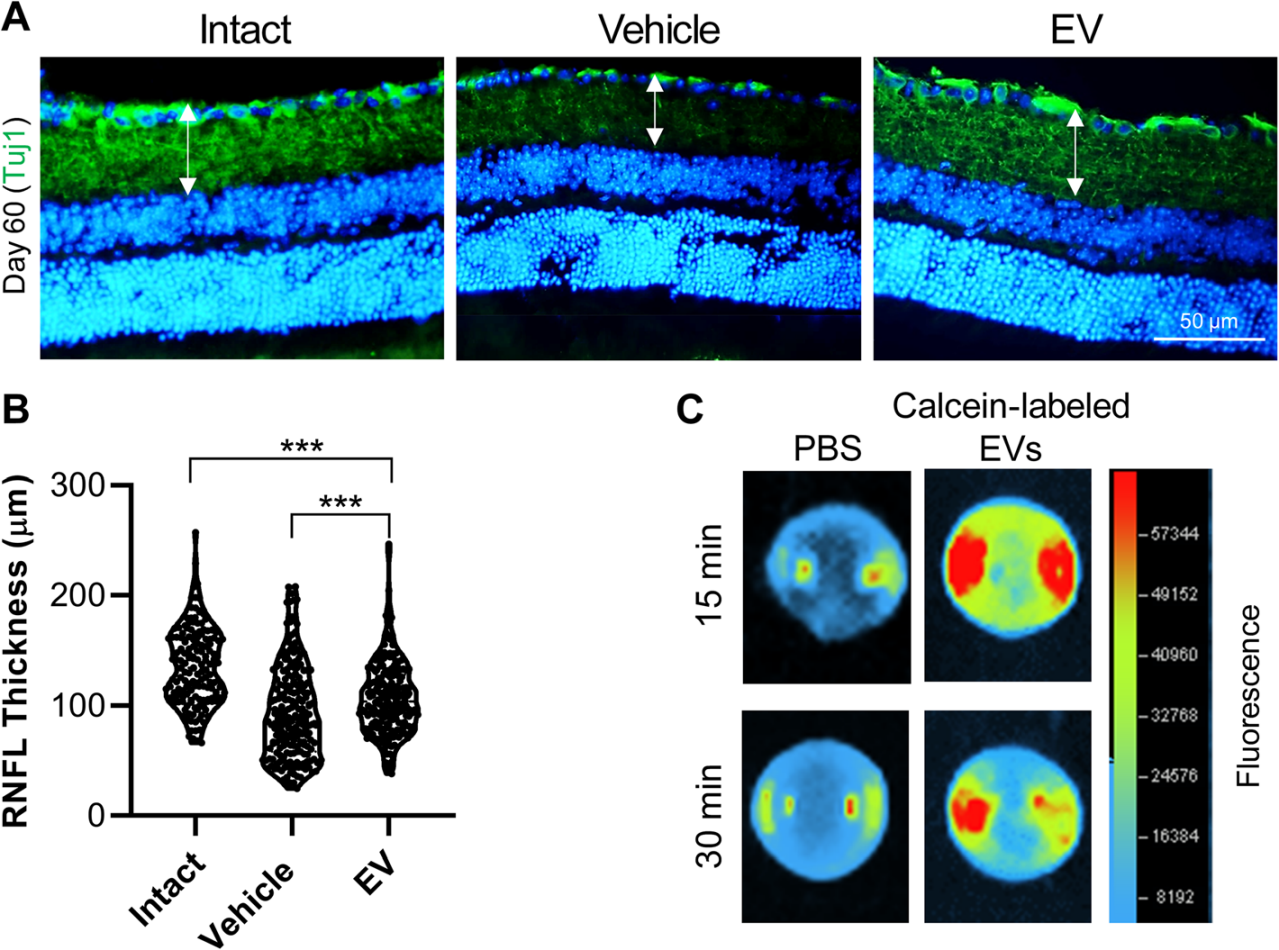
Retinal nerve fiber layer thickness (RNFL) 60 days post-treatment. **a** Immunostaining against Tuj1 as a neural marker. The nuclei were counterstained with DAPI. **b** The diameter of the Tuj1+ area revealed by immunostaining in (**a**). The vehicle group had a decreased retinal nerve fiber layer (RNFL) compared with the extracellular vesicle (EV) group. Data are presented as Violin plot for four optic nerves per group. The data were analyzed by the un-paired *t* test. ***P < 0.001. **c** Representative image of mice eye from dorsal view, enucleated 15 and 30 min post-injection of Calcein-labeled EVs via tail vein

We also evaluated the thickness of the RNFL by staining for the Tuj1 marker at 60 days post-injury (Fig. 5a). A comparison of the RNFL thickness in whole retina demonstrated improvement after the EV injections (106.19 ± 6.69 μm) compared to the vehicle administration (84.75 ± 13.46 μm). However, the difference between the EV and intact groups was not statistically significant (Fig. 5b). Tracing of the EVs with Calcein demonstrated the vitreal accumulation of EVs, post-injection. However, we could not find MSCs in the sections of the retina by immunostaining for human specific antibody for TRA-1-85 (Supplementary Fig. 1). Generally, these data demonstrated the ability of EVs to protect RGCs or regenerate and/or through both effects.

### Extracellular vesicle (EV) treatment suppresses the tauopathy process and ameliorates *cistauosis* in degenerating retinal ganglion cells (RGCs) upon optic nerve crush (ONC)

**Fig. 6:**
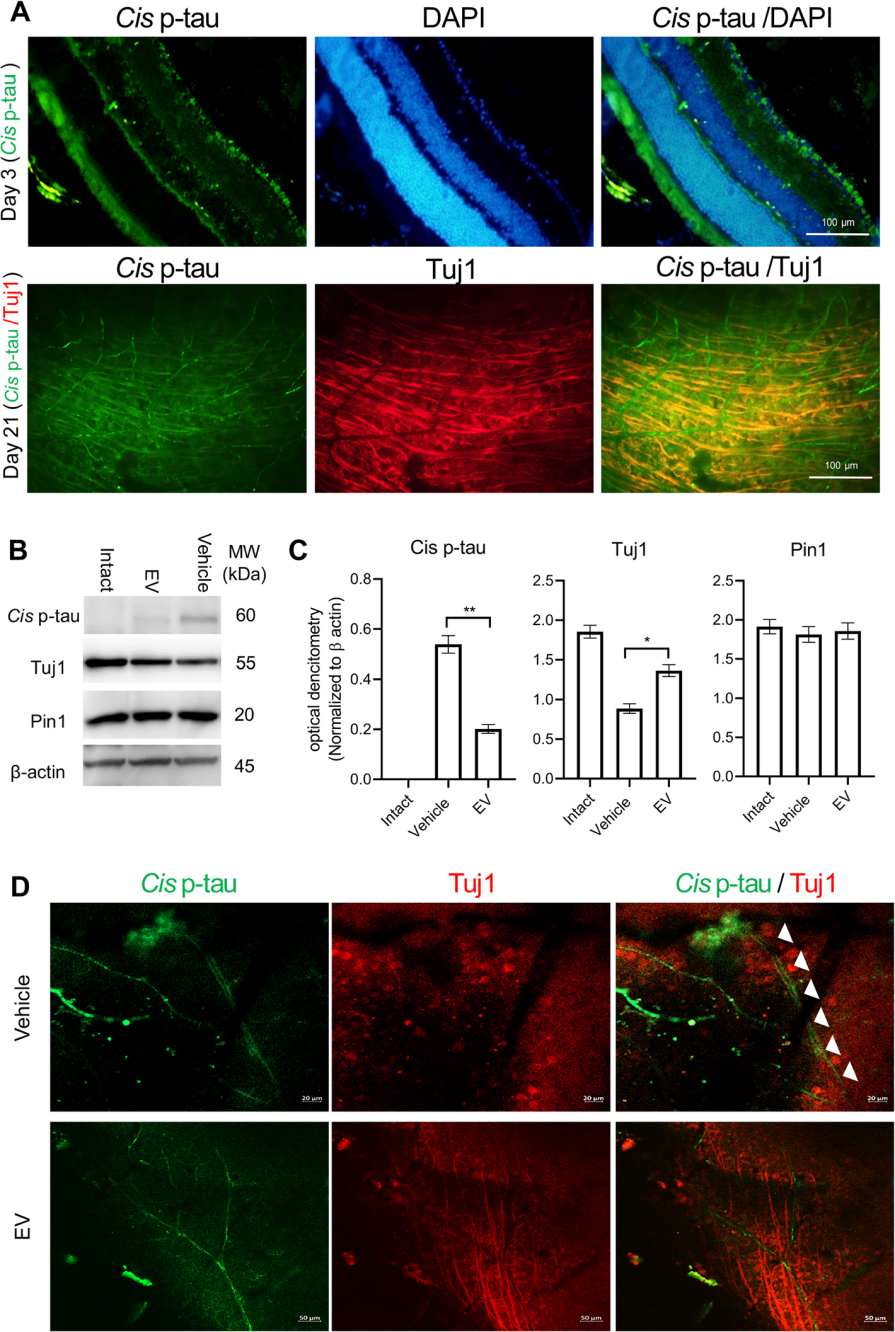
Cistauosis in retinal ganglion cells (RGCs) post-treatment. **a** On day 3, we observed obvious signs of *cis* p-tau formation in the retinal ganglion cell (RGC) layer of the injured retina. At 21 days post-injury, double-stained *cis* p-tau/Tuj 1 showed numerous axons that contained cis p-tau, while it was also observed in the retinal vascular system. **b** Western blot of *cis* p-tau, Tuj 1, pin 1, and β-actin. **c** Optical densitometry of cis p-tau, Tuj1, pin1 which has normalized to β-actin. An increase in *cis* p-tau resulted in decreased Tuj1 levels and vice versa. Data are given as mean ± SD for four samples per group. The data were analyzed by the un-paired *t* test. *P < 0.05, **P < 0.01. **d** Single laser confocal plane shows the degenerating double-positive *cis* p-tau and Tuj1 neurons observed in the vehicle group 60 days post-treatment. The axons in the extracellular vesicle (EV) group are absent in *cis* p-tau

The immunostained retinal sections with *cis* p-tau antibody demonstrated prominent *cis* p-tau in those crush models. However, the EV injections reduced *cis* p-tau levels and blocked the tauopathy process. There were no *cis* p-tau+ sections in those crush models at 6 and 12 h after the trauma. There was *cis* p-tau accumulation at 24 h, which increased by day 3 (Fig. 6a). In this time after the injury, we observed *cis p-tau* exclusively in the RGC cytoplasms while they were limited to only the RGC layer. To examine *cis p-tau* localization, the sections and whole injured retinas were double stained with Tuj 1 and *cis* p-tau. Our findings at 21 days post-injury showed that *cis* p-tau formed in the inner nuclear and RGC layers in addition to the vasculature, but not in the photoreceptor cells (data not shown). Data from whole mount co-stained with Tuj 1/*cis* p-tau confirmed the presence of *cis* p-tau in the injured RGC axons (Fig. 6a). As shown in Fig. 6b and c, there were no significant differences in Pin1 expression levels in the three groups (Fig. 6b, c and d), while there was an obvious inverse relationship between Tuj1 and *cis* p-tau. We examined axonal degeneration after *cis* p-tau formation at 60 days post-crush. We observed degenerating axons that positively stained with *cis* p-tau in the vehicle group, but not the EV group (Fig. 6e).

## Discussion

The therapeutic potential of somatic tissue-derived MSCs has been demonstrated in animal models of ONC. It has been shown that MSCs have a neuroprotective and regenerative effect [5–9]. The ONC model of a CNS injury is characterized by RGC death and the inability of axons to regenerate. Therefore, RGC are neither replaceable nor capable of axon regeneration. In this study, we observed significant, sustained neuroprotective and axogenic effects, in addition to preservation of retinal function, after injections of MSCs and EVs. Compared to somatic tissue-derived MSCs, ES-MSCs showed faster proliferation and express lower levels of inflammatory cytokines and capability of immune modulation [15, 24, 26, 27]. Furthermore, ES-MSCs show an increased growth rate during early in vitro expansion [24, 26, 27] and overcome the limitations of harvesting MSCs from adult tissues that include the availability of suitable donors, invasive procedures, limited number of cells obtained during the harvesting process, and restricted in vitro expansion capacity. PS-MSC have a high immunomodulatory effect [15, 26], which may related to their high secretions of anti-inflammatory cytokines, TGF-β and IL-10, and low production of pro-inflammatory cytokines such as IFN-γ [15, 56, 57].

Secreted neurotrophic factors such as platelet-derived growth factor and brain-derived neurotrophic factor are important for neuroprotection of RGCs [31, 58]. Other secreted factors, such as Wnt3a, have been implicated in the neuroprotective effect of MSCs on CNS neurons [35].

Although PS-MSCs have proven therapeutic benefits [16–26] and they have potential for differentiation into neural-like cells, however, the generation of functional neuronal cells from a somatic MSCs did not report. Moreover, several evidences strongly suggest the dominant mechanism of action of these cells is a paracrine-mediated effect with secreted factors and it is easier to use their cell-free active components. EVs can be isolated relatively easily; they benefit paracrine repair without the risks [59], are easy to store, and do not proliferate. These qualities are important for the generation of a cell-free therapy. The reports have demonstrated that the therapeutic effect of EVs were reliant both on proteins and miRNA [32]. Here, we have shown that ES MSC-derived EV were of benefit for RGC survival and retinal function without a loss of efficacy. The therapeutic efficacy of these MSC-EVs has been demonstrated in models of experimental autoimmune uveitis [33], ischemia [34], glaucoma [35, 36], ONC [37, 38], and light injury of retinal pigmented epithelium [39, 40]. While MSC-EVs have demonstrated therapeutic effects, the fibroblast-EVs do not have any significant effects on neuroprotection and neuritogenesis [35, 38].

It is important to recognize that our ES-MSC EVs were injected at 2, 4, and 6 days post-ONC as opposed to previous studies that performed a single transplant of MSCs [5–9] and EVs [33–36, 39, 40] on the day of surgery. Two previous studies have similarly delivered three injections in 3 weeks BM MSC-EVs in an ONC model [35, 37, 38]. We chose this treatment regime to partially emulate the continuous secretion of ES-MSC-derived exosomes. It has been demonstrated that RGC are not the only target of MSC-EVs [38]; thus, it is not clear if the therapeutic effect we observed was via a direct effect on the RGC or through retinal intermediaries.

The significant neuroprotection afforded by ES-MSC EVs was corroborated by our decision time data through the visual behavioral test, which demonstrated significant protection of RGC axons measured as RGC survival, retrograde and anterograde tracing tests, and RNFL thickness in addition to RGC axon regeneration demonstrated by in vivo GAP-43 expression. The effect of BM MSC-EVs on neurite outgrowth has been reported in vitro [38] and in vivo [60, 61].

Additionally, we demonstrated that EV treatment suppressed *cis* p-tau accumulation, as an early driver of tauopathy and the neurodegeneration process [47–49]. We found a remarkable increase in acute *cis* p-tau levels after the crush. This, in turn, disrupts the axonal microtubule network, spreads to other neurons, and leads to apoptosis, which is a process termed “*cistauosis*”. Notably, we observed *cis* p-tau 24 h after the crush, but not at the earlier time points. From day 3 onward, there was a significant increase in *cis* p-tau, as we observed prominent *cis* p-tau accumulation in whole retina Tuj1+ neurons on day 21 post-injury. The increased *cis* p-tau level corresponded to the Tuj1 decrease, which demonstrated retinal *cistauosis*. Also, we observed prominent neurodegeneration in the untreated group. Interestingly, EV treatment could heal the RGC degeneration, which likely occurred via amelioration of *cis* p-tau*.*

## Conclusion

Taken together, human ES-MSC and ES-MSC-derived EVs promote neuroprotection and functional preservation of RGC in an ONC mice with rescue of tauopathy process. Ease of isolation, storage, and transplantation without the complication and risks related to cell transplantations makes ES-MSC a good candidate as an adjunctive therapy to RGC degeneration. This would provide a potential future treatment for optic nerve repair secondary to traumatic and compressive. EV may provide an off-the-shelf resource in appropriate time for treating degenerating RGCs. However, these findings are limited due to using of one human PS cell line for generating MSC and EV. Further studies to determine the ES-MSC EV effective molecules and their targets for optimization and translational purposes and more human PS cell line-derived MSC and EVs are needed. In translating to the clinic, some issues such as treatment dosage, administration route, and immune compromised subjects remain to be clear.

## Supplementary information

**Additional file 1 : Supplementary Fig. 1.** Immunostaining of retinas for STEM 121 as specific human cell marker on MSC injected mice. No human cells demonstrated in central and peripheral parts of the retina at 21nd days post injury.

**Additional file 2 : Supplementary Table 1.** Antibodies used in this study.

## Data Availability

All data generated or analyzed during this study are included in this published article.

## Abbreviations

BN: Bone marrow
BSA: Bovine serum albumin
CD marker: Cluster of differentiation marker
CTB: Cholera toxin B
DLS: Dynamic light scattering
ECL: Enhanced chemiluminescence
ES cell: Embryonic stem cell
ES-MSC: ES cell-derived MSC
EVs: Extracellular vesicles
FBS: Fetal bovine serum
GAP43: Growth-associated protein 43
HRP: Horseradish peroxidase
iPS cell: Induced pluripotent stem cell
MEM: Minimal essential medium
MSC: Mesenchymal stem cell
OD: Optical density
ONC: Optic nerve crush
PBS-: Phosphate-buffered solution without calcium and magnesium
PFA: Paraformaldehyde
Pin1: Peptidyl-prolyl *cis*/*trans* isomerase 1
PS cell: Pluripotent stem cell
PVDF: Poly vinylidene difluoride
RGC: Retinal ganglion cell
RNFL: Retinal nerve fiber layer
SEM: Scanning electron microscopy
TSG101: Tumor susceptibility gene 101
