# Supplementary figures and images for: Extracellular vesicles derived from human ES-MSCs protect retinal ganglion cells and preserve retinal function in a rodent model of optic nerve injury
Source: Stem Cell Res Ther. 2020 May 27;11:203. doi: 10.1186/s13287-020-01702-x (PMC7251703; doi:10.1186/s13287-020-01702-x)

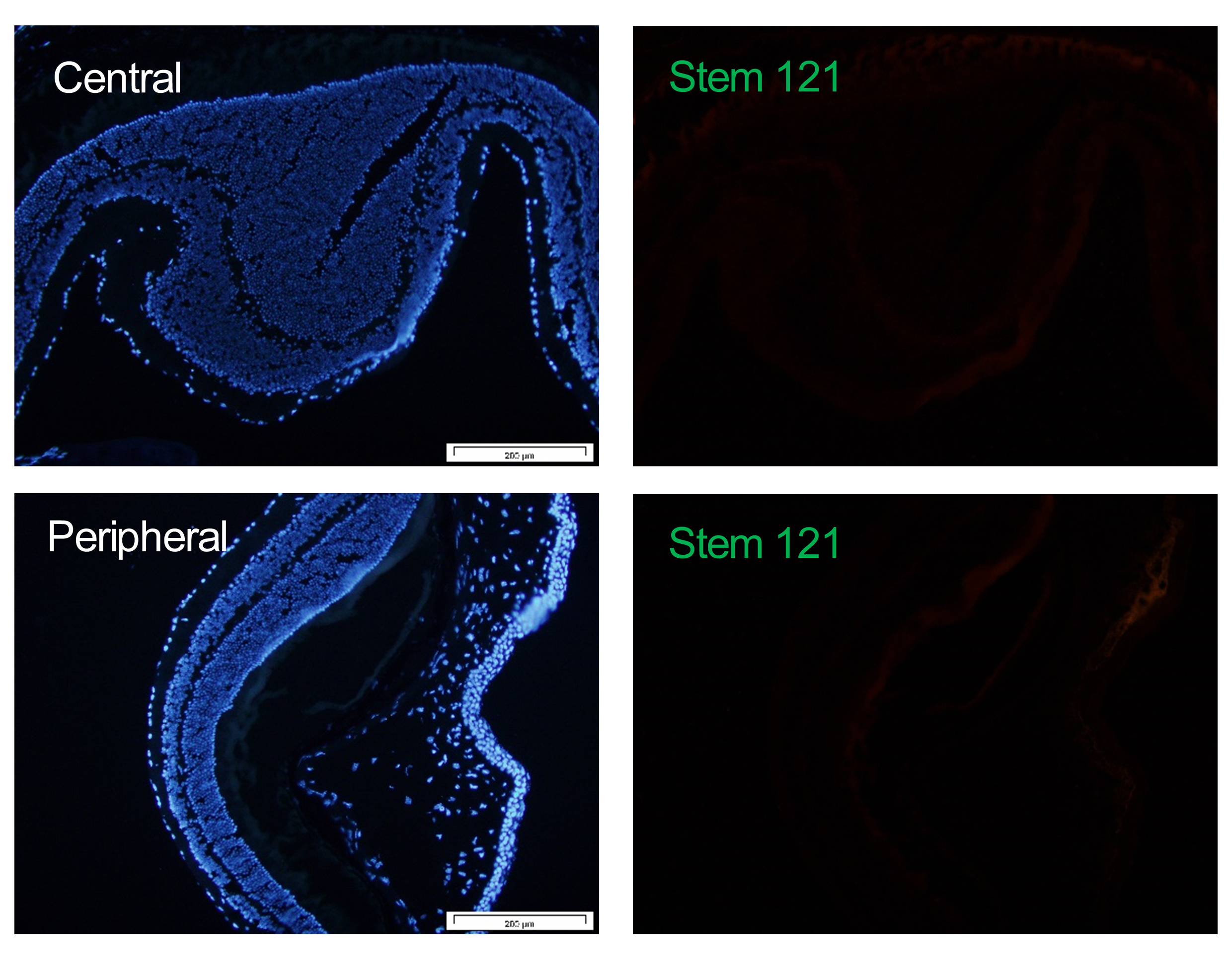

Supplement: Supplementary file 1 — Additional file 1 : Supplementary Fig. 1. Immunostaining of retinas for STEM 121 as specific human cell marker on MSC injected mice. No human cells demonstrated in central and peripheral parts of the retina at 21nd days post injury. [file 13287_2020_1702_MOESM1_ESM.tif]
