# Supplementary material for: Extracellular vesicles derived from human ES-MSCs protect retinal ganglion cells and preserve retinal function in a rodent model of optic nerve injury
Source: Stem Cell Res Ther. 2020 May 27;11:203. doi: 10.1186/s13287-020-01702-x (PMC7251703; doi:10.1186/s13287-020-01702-x)
Supplement: Supplementary file 2 — Additional file 2 : Supplementary Table 1. Antibodies used in this study. [file 13287_2020_1702_MOESM2_ESM.docx]

**Supplementary Table 1:** Antibodies used in this study.

| Antibody (Ab) | Company | Catalog no. | Dilution rate | |
| --- | --- | --- | --- | --- |
| Primary Ab |  |  | Immunostaining | Western blot |
| Brn3a | Santa Cruz | SC-31984 | 1:200 |  |
| Tuj1 | Abcam | 18207 | 1:100 | 1:10 000 |
| GAP43 | Invitrogen | 33-5000 | 1:200 |  |
| Pin1 | Santa Cruz | SC-46660 |  | 2:10 000 |
| β-actin | Proteintech | HRP-60008 |  | 1:20 000 |
| CD63 | Santa Cruz | SC-13118 |  | 1:500 |
| CD81 | Santa Cruz | SC-7637 |  | 1:500 |
| TSG101 | GeneTex | Gtx70255 |  | 1:500 |
| Calnexin | Abcam | Ab66332 |  | 1:500 |
| Stem 121 | Takara Bio | Y40410 | 1:200 |  |
| Secondary Ab |  |  |  |  |
| [Alexa Fluor® 568 Donkey Anti-goat (IgG)](https://www.abcam.com/Donkey-Goat-IgG-HL-Alexa-Fluor-568-preadsorbed-ab175704.html) | Abcam | Ab175704 | 1:500 |  |
| [Alexa Fluor® 488 goat Anti-mouse (IgG)](https://www.abcam.com/Donkey-Goat-IgG-HL-Alexa-Fluor-568-preadsorbed-ab175704.html) | Invitrogen | A-11001 | 1:500 |  |
| [Alexa Fluor® 488 goat Anti-rabbit (IgG)](https://www.abcam.com/Donkey-Goat-IgG-HL-Alexa-Fluor-568-preadsorbed-ab175704.html) | Invitrogen | A-11008 | 1:500 |  |
| Rabbit anti-mouse IgG antibody, HRP conjugate | Sigma-Aldrich | AP160P |  | 1:100 000 |
| Goat anti-rabbit IgG antibody, HRP conjugate | Abcam | ab102287 |  | 1:50 000 |
